# Machine learning in medicine: should the pursuit of enhanced interpretability be abandoned?

**DOI:** 10.1136/medethics-2020-107102

**Published:** 2021-05-18

**Authors:** Chang Ho Yoon, Robert Torrance, Naomi Scheinerman

**Affiliations:** 1 Big Data Institute, Oxford University, Oxford, UK; 2 Medical Sciences Doctoral Training Centre, Oxford University, Oxford, UK; 3 Nuffield Department of Population Health, University of Oxford Richard Doll Building, Oxford, UK; 4 Faculty of Public Health and Policy, London School of Hygiene and Tropical Medicine, London, UK; 5 Department of Medical Ethics and Health Policy, Perelman School of Medicine at the University of Pennsylvania, Philadelphia, Pennsylvania, USA

**Keywords:** clinical ethics, decision-making

## Abstract

We argue why interpretability should have primacy alongside empiricism for several reasons: first, if machine learning (ML) models are beginning to render some of the high-risk healthcare decisions instead of clinicians, these models pose a novel medicolegal and ethical frontier that is incompletely addressed by current methods of appraising medical interventions like pharmacological therapies; second, a number of judicial precedents underpinning medical liability and negligence are compromised when ‘autonomous’ ML recommendations are considered to be en par with human instruction in specific contexts; third, explainable algorithms may be more amenable to the ascertainment and minimisation of biases, with repercussions for racial equity as well as scientific reproducibility and generalisability. We conclude with some reasons for the ineludible importance of interpretability, such as the establishment of trust, in overcoming perhaps the most difficult challenge ML will face in a high-stakes environment like healthcare: professional and public acceptance.

## Introduction

The serendipitous intersection of mature theoretical foundations, powerful computational infrastructure and ‘big data’ has impelled the groundbreaking advances of machine learning (ML) that have epitomised what Klaus Schwab of the World Economic Forum famously described as the ‘Fourth Industrial Revolution’ in 2015.^1^

Since its debut in the 1980s, computerised clinical decision support (CDS) has become increasingly integrated into the healthcare ecosystem, striving with varying success to facilitate healthcare professionals with everything from streamlining administrative duties to increasing diagnostic accuracy.^2^ Some view ML to be a natural evolution of CDS, while others highlight it as a revolutionary leap in the ‘intelligence’ of machines, now trainable to the standard of human experts in specific contexts.^3 4^ These recent invasions of a formerly exclusive domain of human minds have garnered as much enthusiasm as they have concerns of transforming the basic professional and moral duties of doctors.^5^ The opacity of these perhaps dystopian ‘black box’ ML models has ignited cries for causal explanations, or alternatively for transparency on how they arrive at answers that may have the power to impact the lives of so many, particularly in the wake of a sobering history of predictive algorithms that have only managed to amplify pre-existing racial inequities in healthcare provision and contributed dangerously to erroneous decision-making in criminal justice.^6–8^ A rapidly burgeoning quest for increasing interpretability (often used synonymously with explainability in ML research) offers the possibility of allaying some of these fears.

Yet the view that increased interpretability is of utmost importance to progress in ML in healthcare is not held by all. Alex John London argues that interpretability in ML may reflect the widely held misbelief that medical expertise is more consistently explicable and axiomatic than it is in reality, arguing that medical decision-making is in fact influenced by an amalgam of the anecdotal, empirical, associationist and causal.^5^ London asserts that replicable validation of any ML model’s efficacy should therefore be emphasised over an intractable requirement of interpretability, and that such interpretability may in certain situations prove deceptive or deleterious.^5^ Recent emphatic invocations to the Popperian ideals of reproducibility in ML align with London’s thesis that empirical validation should be prioritised, raising the question of the extent to which the pursuit of increased interpretability should feature in the research and development of ML models in healthcare. If efforts to shine a proverbial light into the ‘black box’ serve only to satisfy academic curiosity and quell irrational fears, then it could be argued that the considerable time and resources currently devoted to the pursuit of increased interpretability are ethically unjustifiable. To address these concerns, we respond to the following question: should the pursuit of increased interpretability of ML models in healthcare be abandoned?

In addition to the implications of interpretability on establishing trust—an argument that features prominently in the most contemporaneous ethical discussions and guidelines for ML research^9–12^—we discuss the value of interpretability in the context of extant medicolegal precedents as well as its potential value in expediating the iterative process of model development and external validation.

## Equality and equity

Ingrained biases within the data sets and mathematical formulae that train ML algorithms present a pernicious and potentially far-reaching threat to justice, which might remain undetected if interpretability is not pursued. Even relatively ‘simple’, more interpretable algorithms require considerable ingenuity, insight and domain expertise in order to unveil and redress the kinds of prejudices that they may be propagating to the detriment of many vulnerable people. In a recent example of this, Obermeyer *et al* painstakingly uncovered and helped to rectify a proprietary healthcare insurance algorithm applied to millions of Americans every year that was significantly biased against Black Americans.^8^ Although they were able to circumnavigate the opacity of the algorithm with large, granular data sets and a jolt of creativity, their inquiry would have been appreciably easier with a window into the algorithm’s lines of reasoning.

Biases stacked against the under-represented and more vulnerable populations have become especially topical as healthcare systems and practitioners buckling under the unparalleled onus of the COVID-19 pandemic look towards technological solutions, including those with an ML/artificial intelligence (AI) backend, to assist them. As much as these models are in critical need to assuage the pressures of COVID-19, a recent systematic review of 145 prediction models for COVID-19 suggested that the majority of these models were too vulnerable to bias for clinical utilisation and fundamentally lacked model transparency.^13^

With respect to associationist analytical modalities like ML models, some have argued that an awareness of the limitations of what one may deduce from any given data set is more important than the need for model interpretability per se.^5 14^ While such a philosophy would discourage the mistaken conflation of correlation with causal inference, for example, some intrinsic shortcomings of the data sets may remain latent until post hoc reflection, a vital phase of education, whether in human or machine. In a series of experiments exploring the utility of algorithms to explain other ML models, Tulio Ribeiro *et al* reference a classic anecdote of specious learning, where a model that can perfectly segregate images of huskies from wolves may be trusted to continue doing so until the ‘black box’ is revealed to be merely seeking the presence of snow in the background to ‘correctly’ classify a wolf.^15^ In other words, the standard metrics of gauging performance (such as classification accuracy and positive predictive value) would have served only to cloak the ill logic of the model with a flattering veil. By seeing using the machine’s ‘eyes’ via a human-interpretable lens, the corrigible biases of the data set came to light; with empirical validation alone, on the other hand, they may have not.

Drawing parallels from the way we as humans find it more difficult, if not impossible, to learn from a ‘wicked’ learning environment—where informational mismatches and ‘black boxes’ are rife—achieving interpretability in ML models is likely to assist substantially in discerning and correcting for the very injustices and biases that these models are otherwise quite capable of ‘unintelligently’ enforcing.^16^

## Reproducing interpretability and accuracy

In conjunction with the problematic biases of data sets and their algorithmically distilled abstractions, ML research has been reeling in the wake of another bias: a reproducibility crisis fanned by publication bias, similar to that seen in other branches of science.^17^ The usual suspects are manifest here—incomplete methodology, protection of intellectual property, inter alia—compounded by more domain-specific challenges from ML’s dependence on a colossal array of ‘experiential’ variables, including the exact data set for training, the hyperparameters ‘tuned’ to optimise performance and the fundamental stochasticity of learning.^17^

The outcomes of healthcare ML models that are most frequently communicated are measures of predictive performance, for example, diagnostic accuracy, sensitivity and specificity. The aforementioned instance of misplaced ‘logic’ in distinguishing wolves from huskies is a fable of relevance here too: two ML models may be just as precise as one another, but the ‘rationale’ of one model may be entirely ungeneralisable (using snow in the backdrop as the sole determinant of wolves would prohibit the model from sighting them in any other context). Some have argued that these discrepancies may not matter as long as more clinically meaningful outcome measures, such as mortality and morbidity, have been robustly and reproducibly assessed. However, with the aid of error auditing through interpretability methods, in silico research and development cycles can avail themselves of far superior efficiencies in cost and progress than their counterparts in, for instance, the pharmaceutical industry; this argument in support of interpretability may equally apply to the inherent inefficiencies observed with recent, siloed developments of COVID-19 diagnostic and prognostic models.^13^

Error auditing does not simply serve the purpose of correcting underperforming, biased and/or ungeneralisable models, but it could also help engender trust in the use of ML in healthcare (see the Clinical adoption section). It is widely believed that ethical governance is key, although insufficient by itself, to building trust in AI, championing ‘aircraft flight data recorder’-type post hoc analyses to shed light on contributors to adverse events involving the use of AI.^9–12 18^

While error auditing should not replace rigorous preclinical development phases that can unequivocally overcome some of the reproducibility concerns outlined above, it can be argued that error auditing has an important, complementary role to play in promoting the safe application of ML in healthcare, aided by enhancing interpretability.

One of the reverberating arguments for prioritising empirical validation over interpretability is the commonly held belief that interpretability is a counterpoise to predictive performance. In the field of genomics, for example, within a few years of Libbrecht and Noble’s influential overview of genetic ML approaches, which echoed the *zeitgeist* of interpretability at the expense of performance, significant strides have been made in ameliorating interpretability along with predictive power.^19 20^ Furthermore, as the complexity of ML models has increased, the cross-disciplinary integration of other computer science fields, like visual analytics, is starting to beget more human-interpretable visualisations of what these models are ‘learning’. Continuing along the genomic theme, the interpretability of ML models has benefited from ideas jumping across disciplinary boundaries: Koo and Ploenzke improved the interpretability of a genomic ML model by exploiting a method frequently used to separate true signal from background noise in digital microscopy.^21^ This sort of work lends weight to the importance of cultivating more collaboration between ML researchers and experts in related computer science fields to ultimately spawn more visually understandable representations of what ML models are ‘learning’, particularly (again) when applied to a medical realm that holds such deep significance to individuals and groups as genomics.^22^

## Accountability and the law

As ML-driven healthcare applications become more sophisticated, they can be increasingly considered to be an extension of the routine cognitive processes of live physicians. The most advanced ML healthcare applications today are able to stand in for doctors with marginal human oversight, as exemplified by the diagnostic accuracy of an offline AI smartphone app in screening for diabetic retinopathy.^23^ By providing ophthalmological ‘expertise’ in regions underserved by existing healthcare infrastructures, these computer programs are acting *en lieu* of their human counterparts. In this sense, ML in healthcare stands distinct from more conventional ‘interventions’ assessed in clinical trials, such as pharmacotherapy and medical devices, with ML models capable of decision-making themselves.

By outsourcing some of the decision-making to ML models, the question arises as to who is accountable if ML model failure results in an adverse event. In AI literature, this question has been exhaustively discussed, not least with regard to self-driving cars and autonomous drones. This debate has largely revolved around two categories of issues, which can be crudely described as human versus machine accountability and human versus human accountability. With regard to the former, the question that has been the focus of debate is whether a redistribution of accountability from humans to machines occurs as a result of advances in AI, with a subsequent reduction in ‘meaningful’ human accountability resulting in an ‘accountability gap’.^24^ On the other hand, the latter concerns the question of which human actors are accountable (and to what extent) for an adverse event involving AI when multiple human actors are involved, known as the ‘problem of many hands’.^25^

When seeking to address the question of whether the pursuit of greater interpretability is key to progress in ML in healthcare, one might ask the question of whether interpretability impacts on these accountability debates. As a thought experiment, one might compare two scenarios in which an adverse patient event has occurred (eg, an ML model has misdiagnosed COVID-19 as a common cold, sending the patient home when in fact they required hospitalisation and ventilatory support) as a result of faulty ML ‘reasoning’ despite empirical validation (ML model found to be better than alternatives, and seen to be geographically generalisable from one area to another), with the only difference between the scenarios being the level of interpretability. Suppose that, in scenario 1, the ML model is completely uninterpretable to the attending physician and, in scenario 2, the ML model is fully interpretable and the ML ‘reasoning’ is fully understood by the attending physician. One could then ask the question of whether the distinction between scenario 1 and scenario 2 (level of interpretability) impacts the degree to which the attending physician is accountable for the adverse patient event. One response could be that in scenario 2 the attending physician has full understanding of the illogic of the ML model, thus the decision to proceed with a problematic model despite empirical validation is at the very least risky. The physician is accountable for the adverse outcome, at least to some extent, and should receive a significant proportion of the blame relative to other actors (either human or non-human). In scenario 1, it could be argued that the proportion of responsibility would be different from that in scenario 2, as the physician did not know that the ML model’s reasoning was problematic, in which case the other actors (model developers and empirical validators) may be relatively more accountable compared with those in scenario 2 (if accountability is understood as a zero-sum game). However, regardless of attribution of accountability, if an awareness of the illogic of the ML model in scenario 2 increases the responsibility of the attending physician for the adverse patient outcome, then it must be the case that the physician is relatively less accountable for the adverse patient outcome in scenario 1. If this is the case, with the degree of interpretability constituting the only difference between these two scenarios, one must surely conclude that interpretability of ML models is relevant to accountability.

The question that would then follow would be whether or not current medicolegal benchmarks for determining accountability of healthcare professionals in medical cases, such as the Bolam test, objective patient standard or subjective patient standard, are sufficiently sensitive to accommodate this distinction, or if the application of ML in healthcare behoves consideration of novel or adapted standards that are sensitive to the ethical relevance of interpretability.^26–29^

A legal case from 2018 serves to portray existing accountability structures in regard to the clinical decision support (CDS) tool itself or the misuse thereof.^30^ The case pertained to a young woman who approached her doctor to help her lose weight. Her doctor prescribed a CDS-recommended, yet nonetheless medically contraindicated, prescription of two weight loss medications, an action that cost the patient her life. In such a scenario, does liability rest with the CDS product and its developers, or is this medical malpractice? During the ensuing trial, a medical expert opined that the doctor should be held accountable for the deviation from the expected standard of care, namely the failure to act judiciously as a learned intermediary between the CDS product and the patient. However, a summary judgment was initially granted in favour of the prescribing doctor and the relevant software company, with the medical expert being deemed insufficiently versed in computer programming. In other words, the spotlight was on the software and not on its use (the trial’s conclusion was eventually successfully appealed). Whether the program featured AI or not, the proprietary nature of the CDS program would have rendered the investigation of its function significantly more challenging, a characteristic that has become all too common in this industry, perhaps in the name of accelerating innovation. Indeed, in 2016, subsection 520(o)(1)E was added to the US 21st Century Cures Act, which was originally enacted to catalyse medical product development, thereby exempting a large swathe of CDS software (including ML models) from rigorous testing procedures associated with categorisation as a ‘medical device’, provided the software ‘enables the health care professional to independently review the basis for each recommendation that the software presents’.^31^ It is not clear if independent review by healthcare professionals requires access to a full understanding of the inner workings of the model or if a partial understanding in some cases could be considered sufficient for independent review. However, it is clear from the amendment that the developers of the model face a reduction in scrutiny if the model is interpretable to the healthcare provider, suggestive of a shift in relative accountability for an adverse outcome from the model developer to the healthcare professional. The verdict of the aforementioned trial, for instance, has since been appealed for a similar repositioning of responsibility towards the healthcare professional as the learned intermediary, duty-bound to protect patients from potentially harmful products. However, if confusion about accountability underscores contemporary medicolegal cases like this one, the hypothetical deployment of autonomous AI-based software, sophisticated enough to be less ‘decision support’ and more the primary ‘decision maker’, will conjure many more questions than answers, blurring the existing boundaries between product liability and medical malpractice. For one, how the learned intermediary doctrine would apply in the case of an inherently uninterpretable ML model remains to be mooted.

## Clinical adoption

Empirical validation alone will not necessarily translate into clinical application, for which a *sine qua non* is adoption by healthcare practitioners, a potentially fraught process for any novel technology lacking a homologous precedent. The process of trialling and implementing new drugs is so standardised and normalised in the medical profession that the paucity of expository mechanisms does not preclude their prescription. ML models are, by virtue of their nascency, very distant from this position of interventional ‘privilege’, and for good reason: healthcare is a risk-based domain, where few things are certain, and where trust is a valuable commodity worth earning.

Many researchers of ML in healthcare argue that inextricably linked to the issue of trust is interpretability, a reflection of one of the cardinal responsibilities of medical practitioners: explanation. Explanation lies at the heart of transparency, multidisciplinary discussion, medical apprenticeship and, perhaps, most importantly of all, the patient–clinician dyad that drives life-altering healthcare decisions.

Presently, randomised controlled trials (RCTs) are considered to be the acme of evidence-based medicine. Innumerable medical therapies in circulation today have been justified through such experiments with or without knowledge of their underlying biochemical or biophysical mechanisms. The semantics of ‘causality’ in the setting of RCTs can be misconstrued: indeed, why a certain treatment is being recommended is clearly different from why it works, or why a certain genetic variant may be associated with a particular diagnosis without established knowledge of its pathogenic role. While these minutiae are not mutually exclusive, it is the rationale for why a treatment is recommended that is most commonly integral to ‘shared decision-making’ between patients and their clinicians, offering support for London’s thesis that empirical validation is of paramount importance.^5^

Yet in the case of ML in healthcare, there is reason to believe that if patients or their practitioners are unable to comprehend why an ML algorithm recommends a certain action, trust may never be established. Recent evidence from the medical arena corroborated the pertinence of case-specific explicability for procuring the trust of the physicians at the interface between their patients and the ML model’s proposals; case-by-case rationales were deemed more trustworthy than high-level generalisations of the model’s functionality, a finding that is congruous with the individually tailored care and explanations that are typical of patient–doctor dialogues.^32^

By enabling the users to detect biases and privacy violations, debug appropriately and audit the robustness of any given model’s predictions, trust may be engendered through a more transparent feedback system, which does not need to elaborate on the inner workings of the ML model as much as communicating why certain conclusions or recommendations were drawn. Going forward, the degree of interpretability (hence clinical usability) seems likely to be as decisive as empirical validation in determining the reception and implementation of ML models in medicine.

## Conclusions

ML offers truly unprecedented diagnostic and prognostic opportunities as medicine endeavours to become more personalised and precise than ever before. In both the research and eventual clinical deployment of ML in healthcare, there are multiple ethical and judicial precedents that would be better served if the growing body of ‘empirical validation’ studies were to prioritise, publish and share interpretable ML models. By emphasising interpretability alongside context-specific empirical validation, we may maximise our chances of rectifying deeply entrenched racially and socioeconomically derived health inequities, and earn the trust of those who may benefit the most; but if we are to achieve this, interpretability cannot remain second fiddle to empirical justification.

## Data Availability

There are no data in this work.
